# Age-Friendly and Climate Ready? The Importance of Climate Change to Age-Friendly Transformations

**DOI:** 10.1093/geroni/igaf122.4319

**Published:** 2025-12-31

**Authors:** Erica Husser, Erin Kitt-Lewis, Grace Behr, Michael McShane, Marie Boltz

**Affiliations:** The Pennsylvania State University, University Park, Pennsylvania, United States; The Pennsylvania State University, University Park, Pennsylvania, United States; Maastricht University, Maastricht, Limburg, Netherlands; The Pennsylvania State University, University Park, Pennsylvania, United States; The Pennsylvania State University, University Park, Pennsylvania, United States

## Abstract

Significant investments in Age-Friendly initiatives demonstrate a growing social commitment to reducing ageism and enhancing quality of life for older adults. Exposure to the health impacts of climate change place older adults living alone at high risk for suffering and mortality. The purpose of this qualitative study was to explore perceptions of the natural environment (importance and concern) and preparation for extreme weather events across the age-spectrum. Researchers in a college of nursing partnered with a mobile clinic and attended eight events in five rural communities. We conducted 41 in-person face-to-face semi-structured interviews with randomly selected participants. Iterative thematic analysis resulted in six major themes: Importance of the Natural Environment, Concerns about the Natural Environment, Experiences with Extreme Weather, Preparing for Weather Emergencies, Sharing and Acts of Care, and Causes and Control. Findings illustrate 1) continuity in the value placed on the natural environment across the age-spectrum, 2) a common perception that “others don’t care enough”, and 3) a desire to share knowledge. Findings can be applied to Age-Friendly initiatives to promote shared benefits of healthy environments, address the misperception that no one cares to help normalize conversations about climate change and preparedness, and provide opportunities for age-integration through program activities, civic events, and the built environment. Integrating climate change into Age-Friendly movements is necessary to keep older adults safe, healthy, and thriving, but also to promote age-integration as a powerful mechanism for building strong communities capable of caring for one another during climate-related disasters.

